# Respiratory Syncytial Virus: A WAidid Consensus Document on New Preventive Options

**DOI:** 10.3390/vaccines12121317

**Published:** 2024-11-25

**Authors:** Matteo Riccò, Bahaa Abu-Raya, Giancarlo Icardi, Vana Spoulou, David Greenberg, Oana Falup Pecurariu, Ivan Fan-Ngai Hung, Albert Osterhaus, Vittorio Sambri, Susanna Esposito

**Affiliations:** 1 Servizio di Prevenzione e Sicurezza Negli Ambienti di Lavoro (SPSAL), AUSL-IRCCS di Reggio Emilia, Via Amendola 2, 42122 Reggio Emilia, Italy; matteo.ricco@ausl.re.it; 2 Canadian Center for Vaccinology, Dalhousie University, IWK Health Centre and the Nova Scotia Health Authority, Halifax, NS B3K 6R8, Canada; bh723616@dal.ca; 3 Departments of Pediatrics, Dalhousie University, Halifax, NS B3K 6R8, Canada; 4 Departments of Microbiology and Immunology, Dalhousie University, Halifax, NS B3H 4R2, Canada; 5 Department of Health Sciences (DISSAL), University of Genoa, 16132 Genoa, Italy; icardi@unige.it; 6 IRCCS Ospedale Policlinico San Martino, 16132 Genoa, Italy; 7 Immunobiology and Vaccinology Research Laboratory and Infectious Diseases Department “MAKKA”, First Department of Paediatrics, “Aghia Sophia” Children’s Hospital, Athens Medical School, 11527 Athens, Greece; vspoulou@med.uoa.gr; 8 Pediatric Infectious Diseases Unit, Soroka University Medical Center, Faculty of Health Sciences, Ben Gurion University, Beer Sheva 8410501, Israel; dudi@bgu.ac.il; 9 Children’s Clinical Hospital Brasov, 500063 Brasov, Romania; oanafp@yahoo.co.uk; 10 Faculty of Medicine Brasov, Transilvania University, 500019 Brasov, Romania; 11 Division of Infectious Diseases, Department of Medicine, Queen Mary Hospital, The University of Hong Kong, Hong Kong SAR 999077, China; ivanhung@hku.hk; 12 Research Center for Emerging Infections and Zoonoses, University of Veterinary Medicine Hannover, 30559 Hannover, Germany; albert.osterhaus@tiho-hannover.de; 13 Unit of Microbiology, The Greater Romagna Area Hub Laboratory, 47522 Cesena, Italy; vittorio.sambri@unibo.it; 14 Department Medical and Surgical Sciences (DIMEC), Alma Mater Studiorum University of Bologna, 40126 Bologna, Italy; 15 Pediatric Clinic, Department of Medicine and Surgery, University of Parma, 43126 Parma, Italy

**Keywords:** RSV, RSV vaccine, monoclonal antibodies, nirsevimab, maternal immunization, lower respiratory tract infections

## Abstract

**Background/Objectives:** Respiratory syncytial virus (RSV) is a leading cause of respiratory infections, particularly affecting young infants, older adults, and individuals with comorbidities. **Methods**: This document, developed as a consensus by an international group of experts affiliated with the World Association of Infectious Diseases and Immunological Disorders (WAidid), focuses on recent advancements in RSV prevention, highlighting the introduction of monoclonal antibodies (mAbs) and vaccines. **Results**: Historically, RSV treatment options were limited to supportive care and the monoclonal antibody palivizumab, which required multiple doses. Recent innovations have led to the development of long-acting mAbs, such as nirsevimab, which provide season-long protection with a single dose. Nirsevimab has shown high efficacy in preventing severe RSV-related lower respiratory tract infections (LRTIs) in infants, reducing hospitalizations and ICU admissions. Additionally, new vaccines, such as RSVpreF and RSVpreF3, target older adults and have demonstrated significant efficacy in preventing LRTIs in clinical trials. Maternal vaccination strategies also show promise in providing passive immunity to newborns, protecting them during the most vulnerable early months of life. This document further discusses the global burden of RSV, its economic impact, and the challenges of implementing these preventative strategies in different healthcare settings. **Conclusions**: The evidence supports the integration of both passive (mAbs) and active (vaccines) immunization approaches as effective tools to mitigate the public health impact of RSV. The combined use of these interventions could substantially reduce RSV-related morbidity and mortality across various age groups and populations, emphasizing the importance of widespread immunization efforts.

## 1. Introduction

Human respiratory syncytial virus (RSV) is a small (150 nm; range 120–300 nm), filamentous and enveloped RNA virus that belongs to the *Orthopneumovirus* genus from the *Pneumoviridae* family in the mononegavirales order [1,2,3] and causes respiratory infections in children and adults [4,5,6]. Usually, RSV infections result in influenza like-illnesses (ILI) including mild, cold-like signs and symptoms [2], but younger infants (i.e., those aged 6 months or less, including those premature and/or small for gestational weight) and adults aged 60 years or older, particularly if reporting any cardiorespiratory comorbidity, are at high risk for developing acute respiratory illnesses (ARI) and lower respiratory tract illnesses (LRTI), including bronchiolitis and pneumonia [7,8]. RSV-associated ARI and LRTI cause high rates of medical consultations, hospitalizations, and even deaths, in low-resource settings from low-to-middle-income countries (LMICs) [9,10], and also in high-income countries [4,11,12,13].

Treatment for RSV infection in all age groups is largely by supportive care with administration of fluids and oxygen supplementation, and by delivering antipyretics and antibiotics when needed, with high direct and indirect costs [11,14,15,16,17,18,19,20,21]. The antiviral drug for RSV, inhaled ribavirin, has been shown to be marginally effective, and glucocorticoids and bronchodilators are limited in reducing morbidity and mortality due to RSV infection [22,23,24]. This landscape was changed 2 decades ago by the introduction of the monoclonal antibody (mAb) palivizumab [25], and most recently by the availability of new mAbs and effective vaccines [26,27,28,29]. This present document has been therefore designed in order to gather and critically appraise the available evidence on RSV preventative options, providing a comprehensive summary to the potential stakeholders and policymakers. Given the global burden and recent advancements in preventive options for RSV, this document was developed as a consensus by an international group of experts affiliated with the World Association of Infectious Diseases and Immunological Disorders (WAidid).

## 2. Virology and Epidemiology of RSV

### 2.1. Basic Virology and Main Antigens of RSV

RSV has a small, single stranded, non-segmented, negative-sense RNA genome (15 to 16 kb) [2,30,31,32] that encodes 10 genes for a total of 11 proteins [32,33,34,35], including three outer surface proteins and eight other proteins (nonstructural proteins NS1 and NS2, regulatory proteins M2-1 and M2-2, the polymerase protein L, the phosphoprotein P, the RNA-binding protein N, and the assembly protein M) [2,30,36,37].

The main viral antigens of RSV are the surface glycoprotein (G, the attachment protein), the small hydrophobic (SH) protein, and the fusion (F) protein [2,31,32].

The G protein has two main functions. On the one hand, it mediates RSV attachment to the host cell by binding surface glycosaminoglycans (GAG), CX3C chemokine receptor 1 [CX3CR1], and heparan sulphate proteoglycans [HSPG]. On the other hand, the G protein is secreted from infected cells, potentially acting as a “decoy” for antibodies [31,32]. The main sequence of the G protein is highly variable: RSV is usually categorized in strain A and strain B according to its phenotype, and within both strains RSV genotypes are then identified according to its sequence [38].

The SH protein is a small protein that is incorporated into the viral particle [2,36]. Albeit highly conserved, and therefore likely associated with some selective advantages, the function of the SH protein remains unclear [36].

The F protein is considered the main pathogenetic factor of RSV [31,32,39], as it mediates the invasion process and contributes to the escape strategy of RSV from the immune system [31,32,34,39]. The F protein interacts with a series of receptors including nucleolin (NCL), epidermal growth factor (EGFR), the receptor of insulin-like growth factor 1 (IGF1R), and the intercellular adhesion molecule 1 (ICAM1) [1,31,32,40]. While the docking of RSV is mainly mediated by the G protein, after its interaction with cellular receptors, the F protein switches from its native or pre-fusion (preF) to the post-fusion (postF) conformation, enabling the fusion of the host and viral plasma membranes, which in turns allows the cellular invasion of viral RNA [31,32,40]. Due to its critical role and to the close connection between structure and function, substantial and still-effective mutations within the sequence of the F protein are considered unlikely. In fact, the F protein is usually highly conserved within RSV strains [41,42,43].

Albeit both the G protein and the F protein (in its preF and postF conformations) elicit neutralizing antibodies (NA), only the F protein stimulates a significant cytotoxic T-lymphocyte response [33,44]. In this regard, the preF hosts more neutralizing epitopes than the postF conformation, thus representing the most suitable target for both vaccines and mAb [44,45,46,47,48].

### 2.2. Epidemiology of RSV

Like other respiratory pathogens such as Influenza Virus and SARS-CoV-2, inter-human transmission of RSV occurs through close contact and via aerosolized droplets [49,50,51]. After its initial replication within the epithelial cells from nasopharynx, the upper respiratory tract, RSV can spread to the small bronchioles or the alveoli of the lower respiratory tract. RSV infection elicits a sustained host immune response with mucus production and tissue inflammation, which from a pathological point of view results into the narrowing of the lower airways, with clinical features of bronchiolitis in young children and acute respiratory illness in older adults or those with underlying medical conditions [2,52,53,54].

Bronchiolitis is a potential complication of RSV infections [55,56,57]. However, the clinical syndromes associated with RSV infection are non-specific. On the one hand, in 2005 Falsey et al. [58] hinted that nasal congestion, dyspnea, and, most notably, wheezing occurred more frequently in adult RSV cases when compared to seasonal influenza A (respectively, 57.63% vs. 41.35%, *p* = 0.03; 80.51% vs. 64.66%, *p* = 0.03; 61.86% vs. 39.85%, *p* = 0.02). On the other hand, the feature that appeared significantly associated with RSV compared to seasonal influenza A was identified in the extensive, often multifocal involvement of lung fields at computed tomography [59]. Therefore, the proper diagnosis of RSV infection requires Real-Time Polymerase Chain Reaction (RT-PCR) assays on respiratory specimens [60,61,62,63]. When not performed, most RSV cases may be missed. In a recent study from Cutrera et al. [64], the retrospective analysis of hospital discharge record database of the Italian Ministry of Health reported a total of 67,746 hospitalizations (time period June 2015–May 2019) with a likely diagnosis of RSV infection. Of them, 40.1% were properly diagnosed and coded as RSV cases, while the majority were cases of acute bronchiolitis not coded as RSV cases but likely to account as RSV cases (59.1%) [64,65].

However, even when RT-PCR is correctly performed, an inaccurate sampling strategy may lead to missed diagnoses [60,61,66,67,68,69]. For example, in the prospective cohort study from Ramirez et al. [68] on 1766 patients hospitalized for ARI in Louisville (Kentucky, USA), RSV was diagnosed in 56 patients by nasopharyngeal specimens, missing nearly half of the patients documented by nasopharyngeal swabs plus additional specimens. Hence, it has been usually postulated that most of the incident cases remain either undiagnosed or misclassified among other viral respiratory disorders [26].

According to available estimates, RSV infections may therefore cause up to 33 million cases of ARI and LRTI every year [4,5,70], with high hospitalization rates [71,72,73,74,75], estimated at around 5.3 hospitalizations per 1000 people per year (95% confidence interval [95%CI] range 4.2–6.8) at a global level [5,6]. However, these figures do not include the outpatient consultations due to RSV infections, whose actual burden still remains very difficult to ascertain at a global level [76]. In the systematic review from Heemskerk et al. [76], the yearly incidence rates for primary care consultations due to RSV in all age categories varied from 0.8 to 330 per 1000 population, and even focusing on high-income-countries estimates remain quite heterogenous due to the heterogenous healthcare policies, leading to the variable accessibility and affordability of primary care. For instance, while a recent retrospective report from Germany hinted at 1.3 to 3.9 million annual outpatient consultations for all age groups from 1998 to 2022, with 43.5% (i.e., 565,000 to 1.7 million) occurring in the age group 0 to 4 years [77], an earlier study from the United Kingdom reported on an estimate of around 350,000 annual medical consultations attributable to RSV in children younger than 5 years [19].

#### 2.2.1. Burden of RSV in Infants and Children

Nearly half of infants are infected by RSV during their first winter of exposure, with resulting high rates of hospitalizations in infants under 1 year of age, peaking between birth and 3 months of age [78]. According to available data from the USA, up to 25% of all hospital admissions among infants < 1 year of age during the winter season are due to RSV [79], with 1 to 2% of all infants being hospitalized in their first winter due to RSV-related conditions [73]. Moreover, RSV has been acknowledged as a leading cause of radiographic pneumonia in the age group 1 to 5 years [80].

Most incident cases usually occur in otherwise healthy infants [4,81] born at their full term [73,76,79,80]. For example, in a recent report from a single center in Italy, among 1262 cases of documented RSV infections, 69.2% occurred in children aged less than 1 year at diagnosis (69.9% of them being aged 3 months or less), and around 75% of cases had no documented pre-existing risk factor. However, there is also considerable evidence that a series of clinical conditions substantially increased the risk for RSV complications, including (but not limited to) prematurity, Down syndrome, congenital heart diseases, chronic pulmonary diseases, neuromuscular disorders, and immunosuppression [64,82]. In fact, patients affected by pre-existing comorbidities [4,5,7,70,83,84,85] can experience high-case fatality ratio (CFR). For example, in a recent meta-analysis, RSV infections occurring in subjects having received bone marrow transplants did result in a CFR of 7.28% (95%CI 4.94 to 10.60) [85].

#### 2.2.2. Burden of RSV in Adults and Elderly

Even though most estimated RSV cases occur in infants aged ≤ 5 years of age [4,5,70], and RSV in adults usually causes only minor symptoms of upper respiratory tract that remains undiagnosed [86], RSV has been increasingly acknowledged as a leading pathogen of older individuals [69,87,88,89,90] where it causes severe respiratory illnesses and LRTI, mostly in older adults [91,92], with high morbidity and mortality [49,58,93]. In fact, substantial lethality associated with RSV in older adults, and particularly among institutionalized ones, has been previously stressed [4,94,95,96], as well as its significant public health impact [20,21,97,98,99].

Through a retrospective analysis of USA mortality and viral surveillance data, in the seminal study of Thompson et al. [100], for the first time, RSV-associated mortality rates in the elderly were documented as even higher than those in children younger than 5 years (i.e., 5.4/100,000 person-years for age < 1 year, 0.9/100,000 person-years for age 1 to 4 years, 29.6/100,000 person-years for adults aged 65 years or older). Similarly, in 2015 the EPIC study included a total of 2340 adults with radiographic evidence of community-acquired pneumonia (January 2010–June 2012), and RSV was documented as the fifth most commonly detected pathogen [101]. More recently, in a cohort study from the USA, RSV was identified in 243 out of 2257 encounters with adults ≥ 6 years old seeking outpatient care for ARI (10.77%); of them, 19% exhibited a severe outcome, with a nearly double the rate in individuals with documented chronic cardiopulmonary disease [102].

In fact, there is substantial evidence that, consistently with data on the pediatric age groups, the total burden of RSV in older adults appears disproportionately higher in subjects with pre-existing cardiopulmonary comorbidities. For example, in the prospective cohort study from Falsey et al. [58], during the time frame 1999–2003 (Rochester, NY, USA), no consultations at emergency departments, hospitalizations, or even deaths were reported from 608 healthy elderly, compared to a rate of 9% for consultations at emergency departments, 16% for hospitalizations, and 5% for RSV-associated deaths in 540 high-risk elderly. RSV infections in the elderly are also associated with long-term substantial changes in their functional status after hospitalization. As documented by Branche et al. [103], up to 14% of the elderly may experience loss of independence at hospital discharge, with 8% facing ongoing loss of independence 7 months after hospitalization. In closed settings such as long-term care facilities, RSV has been associated with even higher attack rates, that in outbreak settings can reach 13.5% over 1 month [104], particularly in subjects with underlying cardiopulmonary conditions.

At a global level, Shi et al. [4] estimated a pooled incidence of 67 cases per 100,000 per year (95%CI 14 to 315), for a total burden of 1.5 million cases among adults aged 65 years or more, mostly occurring in high-income countries. However, as the data from low-income countries and middle-income countries on adult cases of RSV are lacking, these figures are presumptively underestimated [86]. A more recent meta-analysis from Savic et al. [96] hinted at a substantially higher estimate of 162 cases per 100,000 persons (95%CI 84 to 308) for individuals aged 60 years or older, for a corresponding crude number of 5.2 million episodes at a global level. The higher estimates from Savic et al. could be explained by having included a larger share of more recent studies that in turn benefited from improved testing strategies and the more appropriate identification of incident cases [105]. In other words, according to community-based studies, each year 3% to 7% of all the elderly would be affected by at least one episode of RSV infection [86].

Nonetheless, the burden of RSV in older adults could be more effectively appreciated when dealing with outcomes that can be less significantly affected by under-reporting, i.e., hospitalization rates, ICU admission rates, and RSV-associated deaths with their CFR.

#### 2.2.3. RSV Hospitalizations in Adults

Even though the illness associated with RSV in most cases results in mild and self-limiting disease that only in 17 to 28% of cases requires at least one episode of medical assessment, current evidence suggests that in older adults RSV and seasonal, non-pandemic influenza have similar hospitalization rates and disease severity [106]. For example, in the study from Widmer et al. [106], influenza was detected in 33 out of 508 adults aged 60 years or older hospitalized due to ARI in three consecutive seasons, compared to 31 cases of RSV infection (i.e., 6.5% vs. 6.1%). In their retrospective cohort study of nearly 3 million US long-term care (LTC) residents from 2011 to 2017, Bosco et al. [104] reported around 122/100,000 person-years RSV attributable to cardiorespiratory hospitalizations, compared to the estimate of Matias et al. [107] of around 200,000 cardiorespiratory hospital admissions due to RSV in older adults in the USA. More recently, through the retrospective analysis of administrative databases on health claims (i.e., Commercial Claims and Counters, Medicare Supplemental and Coordination of Benefits, and Medicaid Multi-State), Patel et al. [108] estimated that around 5.5% of 301,248 sampled cardiorespiratory hospitalizations were reasonably associated with RSV infection, a proportion that increased to 6.41% when taking into account high-risk adults, while the proportion of hospitalization nominally due to RSV infections and their complications was lower (0.32% and 0.42%, respectively). In a French study from Loubet et al. [91,109] on adults having been hospitalized for respiratory infections shortly before the SARS-CoV-2 pandemic, a total of 17,483 hospitalizations due to RSV were reported, for an estimated rate of 7.2 per 100,000 episodes; interestingly, 56.8% of them were aged 75 years or more, and most of the reported cases (78.6%) had cardiorespiratory and/or metabolic comorbidity. Estimates from the UK have been provided by Fleming et al. [110] for the time period 1995 to 2009, and from Sharp et al. [111] for the time period from 2010 to 2017. Briefly, RSV reasonably caused a burden comparable to that of influenza in both age group 65 to 74 years (hospitalization rate of 71/100,000), and age group ≥ 75 years (251/100,000; 95%CI 186 to 316), with the latter group accounting for the large majority of hospitalizations (around 80%) and reported deaths (around 90%) [110,111].

Actually, Osei-Yeboah et al. [8] have suggested that around 160,000 hospitalizations would occur annually in adults aged ≥ 18 years from countries belonging to the European Union, 92% of which among subjects aged ≥ 65 years, while a recent systematic review identified attack rates ranging from 6.7% to 47.6% and annual incidence rates ranging from 0.5% to 14% for elderly residing in nursing and care homes [112]. Pooled global estimates were provided by the previously referenced studies of Shi et al. [4] and Savic et al. [96], with corresponding rates of 100 and 150 cases per 100,000 persons/years, respectively. Even though available figures consistently suggested that older age groups are at high risk for hospitalizations due to complications of RSV infections, substantial differences have been reported, being reasonably due to the different reporting and testing strategies. As stressed by Kenmoe et al. [90], a main distinction can be identified between HIC and LMIC, with rates ranging in 157 per 100,000 person-years (95%CI 98 to 252) for HIC, and 30 per 100,000 person-years (95%CI 10 to 70) for LMIC. However, even countries with similar socioeconomic status may show quite heterogenous estimates. For example, according to the retrospective analysis of data from the European Respiratory Virus Surveillance Summary platform (ERVISS) recently performed by French Haute Autorité de Santé (HAS) [105], during the RSV season 2023/2024, the proportion of hospitalizations due to RSV-associated ARI for age groups 0 to 4 years ranged from 27 per 100,000 in Belgium to 119 per 100,000 in Austria. Conversely, corresponding hospitalization rates for age group ≥ 60 years were highly comparable, being estimated to 120 per 100,000 in Belgium and 124 per 100,000 in Austria. While data on primary care consultation may be explained by the different involvement of primary care professionals in the management of ILIs and ARIs, the explanation of these findings is much more complicated, possibly relying on the availability of appropriate diagnostic options.

#### 2.2.4. Admission to ICU and CFR Estimates in Adults

The rate of ICU admission due to RSV infections in the elderly was initially estimated as 1.6% by Shi et al. [4], and this estimate has seemingly increased after the COVID-19 pandemic, with estimates ranging from 5.6% to 11% [113,114,115]. According to Savic et al. [96], CFR of RSV-associated LRTI among older adults can be estimated as 7.13% (95%CI 5.40 to 9.36). In this regard, a recent systematic review from Osei Yeboah et al. [112] suggests that RSV cases from nursing and care homes could face even higher CFR estimates, ranging between 7.7% and 23.1%. In fact, all available estimates are affected by high heterogeneity due the sampling strategy of source studies as well as the presence of co-infections.

The documented heterogeneity of available estimates has been presumptively affected also by the increased availability of high-output testing options after the pandemic, and presumptively led to the increased identification of incident cases of RSV, particularly among cases admitted to ICU. For example, the aforementioned study from Loubet et al. [91] reported a CFR equal to 7.3% that increased to 8.1% among individuals aged 60 or more, compared to 9.7% in a single-center study from Recto et al. on 125 adults [116], and to 6.6% (95%CI 5.2–8.2) in a multicenter retrospective study on 1168 adults with documented RSV infections [117]. Notably, in the study from Celante et al. [117], nearly all cases of ICU admissions were characterized by a comorbidity (hypertension, 46%; heart failure, 34%; COPD, 29%; diabetes, 22%; and immunodepression, 29%), with a CFR of 12.8% (95%CI 9.2 to 17.3). In Germany alone, during the 2022/2023 RSV season up to 620,000 outpatients’ consultations and 12,000 hospitalizations did occur in the age group of older adults (≥ 60 years old), with a 16.1% rate of ICU admission (compared to 4.8% in the age group 1 to 2 years), and a CFR of 9.4% [77]. In other words, up to 95% of all RSV-related deaths occurred in age groups ≥ 60 years old [77].

#### 2.2.5. Seasonal Pattern

RSV infections are usually clustered in seasonal epidemics (i.e., “RSV season”), that often overlap with high-circulation seasons of other respiratory viruses, such as influenza and adenovirus [118,119], and more recently even with SARS-CoV-2 [120,121]. In the Northern Hemisphere (including the US, the UK, France, and Germany), RSV season usually occurs during the winter season, beginning in November or December and reaching circulation peak during the winter months of January or February [4,70], ending in March or April and being followed by low rates of new infections during the warm or dry seasons [122,123,124]. However, environmental factors only act indirectly on the spread of RSV. As humans are the only known hosts for RSV [31,32,125], being all new cases forcibly acquired through human contact, RSV season occurs when environmental conditions force individuals in enclosed spaces [126,127,128,129] that in turn favor viral spread through respiratory droplets [130], eventually increasing the likelihood for the inter-human spreading of the pathogen. Not coincidentally, in tropical countries RSV season is rather associated with the hot, humid, and rainy climate of the summer season [127,128,131,132], although it may be present throughout the year [70,133,134], and even in the Northern Hemisphere, RSV circulates year-round in closed settings such as nursing homes, homeless shelters, and refugee settings [49,58,129].

### 2.3. The Impact of the COVID-19 Pandemic

**Figure 1:**
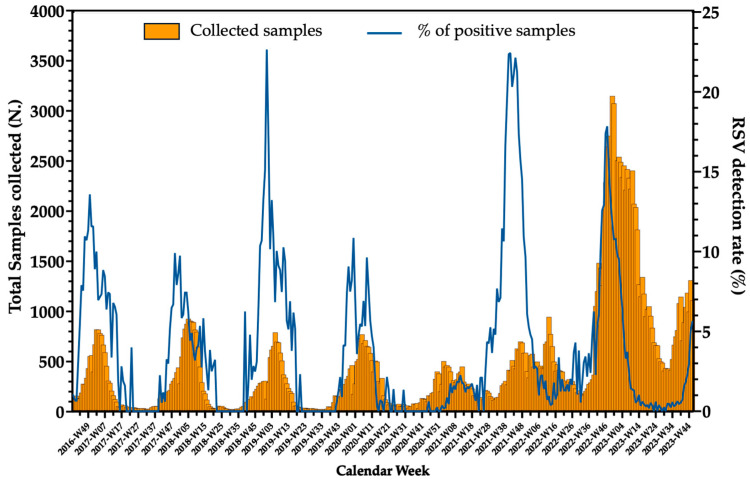
Time trend of respiratory specimens collected since 2016 in EU countries and the corresponding prevalence rate of positive specimens. Original elaboration from data reported by the ECDC Atlas of Infectious Diseases (https://atlas.ecdc.europa.eu/public/index.aspx), accessed on 6 November 2024 [141].

The “first wave” of the SARS-CoV-2 pandemic (i.e., February 2020 to June 2020) impacted on the EU and USA at the end of RSV season, and available data from EU surveillance (Figure 1), and more specifically from Italy, Finland, Belgium, UK, and USA in fact hinted at a sudden end of the epidemic season [135,136,137,138,139]. Even where viral surveillance for RSV was suspended during the low-circulation summer season, available data suggest that substantially no cases were detected during warm and earlier months of the following cold seasons [140].

Non-pharmaceutical interventions (NPIs) taken to mitigate the spread of SARS-CoV-2, such as avoiding crowding and gathering in enclosed spaces, and implementing personal preventive measures, such as mask mandates and improved hand hygiene, directly impaired the transmission cycle of respiratory pathogens, including RSV [142,143]. Interestingly, a similar pattern was documented for several respiratory pathogens, including influenza B/Yamagata, that is nowadays nearly considered extinct [142,143,144]. The subsequent lifting of NPIs further changed RSV epidemiology, leading to an unprecedented peak of RSV infections in the first months following the removal of physical distancing, documenting a sustained circulation of the pathogen [145,146], even in months where it was previously considered rare or unusual [52,142,147], with a peak of the next RSV season [77,147,148], and the extensive involvement of age groups that were considered at relatively low risk for RSV infections and complications. For example, in the recent report from Chen et al. [52], possibly due to the very extensive reliance of the People’s Republic of China National Government on the NPI measures as an alternative to a mass vaccination strategy, a very high circulation of RSV was documented in the summer months, with the proportion of cases among healthy children aged 3 to 5 years increasing from 20.22% in 2019 to 46.53% in 2022 and 34.87% in 2023. Similar results were also reported from other geographic areas, where the seasonality of RSV has remained substantially irregular until 2024 [149]. On the contrary, a recent report from Italy identified earlier seasonality and shorter duration of RSV outbreaks compared to pre-pandemic, still documenting an increased risk of RSV-associated hospitalizations (15.3 cases per 1000 person-years, 95%CI 13.9 to 16.4 in 2021–2022 season, and 19.9 cases per 1000 person-years, 95%CI 19.5 to 21.2 in 2022–2023 compared to 5.6 cases per 1000 persons-years, 95%CI 5.3 to 6.3 in 2019–2020) [150]. Due the progressive and persisting erosion of the RSV seasonality, the number of cases occurring out of season has increased, stressing the public health significance of preventative interventions effective all around the calendar year [151,152].

A likely explanation for this trend could be identified in the limited duration of neutralizing antibodies from respiratory epithelia elicited by natural infection [153,154,155,156,157,158]. As RSV does not elicit long-lasting neutralizing antibodies (NA), NPI could have led to a reduced circulation of the pathogen that in turn let a large population of pediatric patients that remained immunologically naïve to RSV be particularly susceptible to the pathogen during the following seasons [145,146,159,160,161].

Suggestive as they are, the aforementioned estimates should be quite cautiously appreciated. In fact, before the SARS-CoV-2 pandemic, molecular diagnostic options for respiratory infections were more rarely available and widely implemented, particularly in adults [64,77,143,162,163,164]. For example, in a recent report from Lodi et al. [163] on hospitalizations from a single center in Italy, almost no cases of RSV were recorded during the SARS-CoV-2 pandemic, while in the post-pandemic time period, the number of RSV-related hospitalizations showed a +317% increase per season compared to the pre-pandemic time frame.

### 2.4. Economic Burden of RSV

The substantial medical and social burden of RSV is mirrored by its economic burden [20,21,165,166], that has been estimated globally to be approximately 4.82 billion euro for children under the age of 5 years [13]. Notably, the aforementioned estimates are unevenly distributed at a global level, as up to 65% of the estimated costs are in LMIC due to the high proportion of children in high-risk groups. Indeed, RSV represents a resource-consuming condition [20,165,166], particularly for regions with fewer resources, because of high direct and indirect costs [167], the latter due to the productivity losses among caretakers of affected individuals. Global estimates on RSV-associated expenses for patients, relatives, and healthcare systems are, again, quite heterogenous, reflecting the underlying different approaches to healthcare policies. For example, a recent Italian retrospective study did estimate a mean cost for hospitalization ranging from 2007€ to 2617€ [64], compared to mean costs of around 28,586 dollars (standard deviation [SD] 55,523) per episode of RSV-associated LRTI requiring an inpatient stay documented by a national sample of privately insured US children under 5 years of age [168].

Focusing on individuals aged 60 years or more, according to Carrico et al. [167] in the USA alone a total of 4 million RSV cases would lead to a total burden of $6.6 billion in costs (95% uncertainty interval [95%UI], 3.1–12.9). As 94% of this burden is due to direct medical costs, also when dealing with adults only, heterogenous policies and healthcare management strategies could imply highly heterogenous estimates in the economic burden. For example, in 2020 Ackerson et al. [169] identified costs due to hospitalization of adults aged 60 years or more of $16,034 compared to $15,163 for influenza, while in a more recent study from Averin et al. [170], the average costs for the management of RSV cases were estimated to be $42,179 for each hospitalization, $4409 for each consultation in the emergency department, and $922 for each medical consultation. Hospitalizations due to RSV are reasonably associated with increased medical costs also during the follow-up. According to Mac et al. [171], extra costs were estimated to be $28,260 for the first 6 months and $43,721 for the first 2 years after hospital admission (time period 2010 to 2019). Similarly, in a Canadian study from Rafferty et al. [172], the median costs faced by adults hospitalized because of RSV-associated LRTI infections were estimated to be 12,713 Canadian Dollars (around $9500) for the 30 days following the diagnostics to 40,028 Canadian Dollars (around $30,000) in the first follow-up year, an estimate that nearly doubles for subjects aged 80 years or older. Also in the European settings, the costs associated with RSV burden in older adults appear significant, having been estimated to 7215€ for each hospitalization in adults aged 60 years or older, compared to 3335€ in individuals < 18 years [115].

## 3. Preventative Options

Until 2023, the available preventative options for RSV were limited to a small subset of high-risk infants [26,173,174]. In addition, most RSV candidate vaccines did not achieve the desired vaccine efficacy [28,175]. On the other hand, only one mAb targeting the site II of the F protein (i.e., palivizumab) was ultimately licensed and used in real-world settings, and only in high-risk pediatric patients [16,176,177,178]. As palivizumab must be administered monthly for up to a total of five subsequent weight-dependent doses (i.e., 15 mg/kg) [177,179,180], it is associated with high direct and indirect costs [16,181]. For example, Wick et al. have recently estimated that the mean costs of palivizumab per infant who received at least one dose in the first year of life was 5435€ for the birth cohorts 2015–2019 [164]. Therefore, palivizumab was only indicated for a small subset of infants [16,179], and more precisely: (1) children born at 35 weeks of gestation or less and less than 6 months of age at the onset of the RSV season; (2) children less than 2 years of age and requiring treatment for chronic pulmonary disease within the last 6 months; and (3) children less than 2 years of age and with hemodynamically significant congenital heart disease [16,176,177,182].

Since 2023, new preventive options have been offered by the approval of new mAb and vaccines. Available vaccines provide active immunization strategies in older adults and passive immunity to infants by means of maternal vaccinations. Passive immunity of newborn children, achieved either by vaccination in pregnancy or mAb, has the potential to protect young infants given the risk of severe RSV complications (i.e., hospitalizations and even deaths) in the first 6 months of age, when the delivery of a pediatric vaccine would possibly fail to achieve the appropriate immunity [183,184,185], similarly to influenza and pertussis.

In the following sections, the available evidence will be summarized.

### 3.1. Monoclonal Antibodies

New mAbs have been developed in order to overcome the short half-life of palivizumab and to have a longer half-life that persists after one shot through an entire RSV season [186]. Despite its proven efficacy, the development of motavizumab, a second-generation mAb developed from palivizumab, was discontinued in 2010 as still requiring sequential deliveries across the RSV season for retaining its activity against RSV-associated LRTI [186,187]. Suptavumab, another promising mAb targeting site III of preF, was conversely proven as not effective in reducing overall RSV hospitalizations or outpatient LRTI consultations due to RSV infections because of the spontaneous mutation of the active binding site in the dominant RSV group strain [188].

Nirsevimab (Beyfortus, Astrazeneca [Södertälje, Sweden], and Sanofi [Gentilly, France]), was approved by the European Medicine Agency (EMA; October 2022), being also authorized by the UK (11 September 2022), Canada (19 April 2023), and by the US Food and Drug Administration (FDA) (17 July 2023) [189]. Nirsevimab is a long-acting mAb that specifically targets the site ø within of the prefusion F protein [26,190,191]. Since its approval, several real-world experiences from the Northern Hemisphere [192,193,194,195,196,197,198], mostly from Europe and particularly from Spain [197,199], have documented the effectiveness of nirsevimab in preventing severe LRTI [194,196,197]. In an early systematic review with meta-analysis [29], immunization efficacy in real-world settings was estimated to be 90.5% (95%CI 87.1 to 93.0), an estimate that substantially exceeded data from randomized controlled trials (81.0%, 95%CI 71.5 to 87.3), with very limited occurrence of adverse reactions and side effects. Of note, an increase in the median age of hospitalized cases was documented in the University Hospital of Saint-Etienne from 3.5 months (Interquartile Range [IQ] 1.6 to 6.9) in 2022 to 2023 to 6.2 months (IQ 3.4 to 10.6) at the end 2023 [198], and those were infants born before the delivery of this mAb by the local maternity ward.

**Figure 2:**
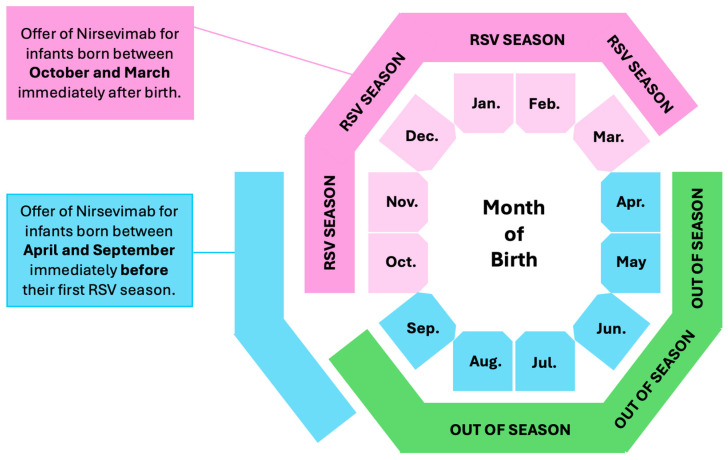
Strategy for the delivery of nirsevimab as recommended by the German Standing Committee on Vaccinations (STIKO) [203].

In a prospective study from Spain including 29,694 children immunized with nirsevimab and 7373 controls not immunized, real-world effectiveness against primary care events during the whole RSV season decreased from 69.0% (95%CI 63.5 to 73.7) during the first month of nirsevimab administration, to 60.9% (95%CI 55.0 to 65.9) in the second month, 50.6% (95%CI 43.6 to 56.7) during the third, and to 37.5% (95%CI 27.6 to 46.1) during the fourth, while the corresponding efficacy against hospitalizations was 93.6% (95%CI 89.7 to 96.1) at 30 days compared to 87.6% (67.7 to 95.3) at 150 days, with comparable estimates for ICU admission (efficacy of 94.5%, 95%CI 87.3 to 97.5 at 30 days, and 92.1%, 95%CI 64.0 to 98.3 at 90 days) [200]. Similarly, in a matched case-control study from France that enrolled all infants younger than 12 months of age who were hospitalized for RSV-associated bronchiolitis in six tertiary hospitals across metropolitan France (15/10—10 December 2023), the effectiveness of nirsevimab against RSV-associated bronchiolitis leading to PICU admission was 69.6% (42.9 to 83.8), compared to 67.2% (38.6 to 82.5) for admissions requiring ventilation support [201]. However, while the efficacy of nirsevimab in children immunized at 15 and 30 days after delivery was highly comparable, it appeared as scarcely effective in children immunized during the fifth month of age (48.3%, 40.9 to 54.9 at the first month) [199,202]. The German Standing Committee on Vaccinations (STIKO) recently recommended the implementation of the immunization strategy that is summarized in Figure 2 [203]. Briefly, nirsevimab could be offered to all infants born between April and September and delivered immediately before their first RSV season. Conversely, children born “in season” could be offered nirsevimab immediately after their birth.

Preliminary data on a Phase 2b/3 study on another long-acting prophylactic mAb, clesrovimab (MK-1654), was recently presented [204]. Similarly to nirsevimab and palivizumab, clesrovimab targets preF protein, but in site IV rather than in site II (palivizumab) and site ø (nirsevimab) [205,206]. The double-blind, randomized, and placebo-controlled RCT MK-1654-004 (NCT04767373) enrolled a total of 3632 healthy preterm and full-term infants from birth to 1 year of age entering their first RSV season who received either a single fixed dose of clesrovimab (105 mg intramuscular injection (IM)) or placebo on Day 1 (T). Preliminary results hint at an immunization efficacy of 90.9% (95%CI 76.2–96.5) in preventing RSV-associated hospitalizations due to LRTI at T + 150 days, and equals to 91.2% (95%CI 77.2–96.6) at T + 180 days, with similar results against episodes of lower respiratory infections medically attended (91.7%, 95%CI 62.9–98.1 for both T + 150 days and T + 180 days) [204]. Interestingly, clesrovimab was developed for being delivered not only during the first RSV season faced by newborns and infants (similarly to nirsevimab and palivizumab), but also during the following season, with an increased dosage (105 mg vs. 210 mg for season 2), providing a new preventative option for high-risk infants during the follow-up season [204,205,206].

### 3.2. Vaccines

Several candidate RSV vaccines are or have been in the development pipeline [26,44,81,87,207,208,209,210,211,212,213,214,215,216,217,218,219,220,221,222,223,224], and several candidate pediatric vaccines are being developed for their use across the various age groups [225,226,227,228,229,230], including:

(1) two protein-based formulates: RSVpreF from Pfizer Inc. (Abrysvo^TM^, Pfizer Europe MA EEIG, Brussels, Belgium), and RSVpreF3 from GlaxoSmithKline LLC (Arexvy^TM^, GlaxoSmithKline Biologicals SA, Rixensart, Belgium) [231,232];
(2) one mRNA formulate: mRNA-1345, from Moderna Inc (mRESVIA^TM^; Moderna Inc., Cambridge, Massachusetts, USA) [233];
(3) two vector-based formulates: adenovirus-based Ad26.RSV.preF from Janssen [234,235,236] that, despite promising results, was temporarily halted in midst of late-stage clinical trials, being only recently relaunched [236], and the poxvirus-vectored vaccine MVA-BN-RSV from Bavarian Nordic A/S (Kvistgård, Denmark), that failed in phase 3, being ultimately discontinued.

To date, only three vaccines were authorized for clinical practice in the USA and/or the EU [232,237,238,239,240]: RSVpreF, RSVpreF3, and mRNA-1273 (Table 1). All of them can be used in adults aged 60 years or older; RSVpreF and RSVpreF3 can be delivered in adults aged 50 years or older if affected by chronic conditions, while only RSVpreF can be delivered in adults aged 18 to 49 if affected by risk factors for RSV-associated infections, and as a maternal vaccination. Conversely, none of them is currently authorized in either children or infants. In the following sections, the main characteristics of available vaccination strategies will be outlined in older adults and as for maternal vaccination.

#### 3.2.1. Adults

Both RSVpreF and RSVpreF3 are subunit vaccines based on the preF protein. Despite the similarity in their antigens, their design has followed a quite different development. In RSVpreF, the bivalent, non-adjuvated formulate includes a similar amount of preF from strain A and strain B RSV (60 µg + 60 µg), while the monovalent RSVpreF3 includes only 120 µg preF from strain A and the AS01 adjuvant [231,232]. Both vaccines should be delivered as a single dose, and the currently approved schedule does not include annual shots. The third vaccine, mRNA-1345, includes a single mRNA sequence (50 µg) that encodes for a stabilized preF protein from strain A RSV, without adjuvant [213,243]. Similarly, the mRNA-1345 schedule currently includes a single dose.

The efficacy of RSVpreF, RSVpreF3, and mRNA-1345 in adults ≥ 60 years was ascertained by a series of RCTs, whose content has been recently reviewed by the STIKO [244], the Advisory Committee on Immunization Practices (ACIP) [233], as well as by Riccò et al. [28], which stressed several substantial shortcomings. First and foremost, all studies were highly heterogeneous in terms of design and assessed outcomes [28,232,237]. While vaccine efficacy for RSVpreF and mRNA-1345 was calculated through the determination of the incident cases of ARI and LRTI, the latter dichotomized in LRTI with two or three or more symptoms [87,213,245,246], studies on RSVpreF3 relied on a quite different case definition of LRTI that included two mild symptoms plus at least one severe symptom [210,241,242]. Second, even reported clinical features were inconsistent across studies in terms of targeted signs and symptoms. On the one hand, RCTs on RSVpreF and mRNA-1345 relied on a panel of five respiratory symptoms (i.e., cough, wheezing, sputum, shortness of breath, tachypnea), two of which (i.e., cough and sputum) are usually associated with upper respiratory tract infections during the early stages of RSV clinical syndrome, irrespective of its eventual severity [26,109,247,248,249]. On other hand, case definition applied in the RCTs on RSVpreF3 [210,241,242] relied on a larger panel of signs and symptoms which included a series of systemic or lower respiratory tract findings. As suggested by STIKO [244] and then stressed by Riccò et al. [28], a LRTI status defined by only two findings from a broader range of signs and symptoms will likely include a high share of subjects only complaining from upper respiratory symptoms like sputum or cough. Conversely, a case definition with three or more findings could be reasonably associated with a more severe syndrome by including signs and/or symptoms such tachypnea/breath shortness or wheezing. Not coincidentally, in their 2023 recommendations [232,237], the ACIP considered to be equal end points in their rationale LRTI with two findings [210,241,242] reported from RCT on RSVpreF3, and LRTI with three or more findings from RCTs on RSVpreF [87].

Second, RCTs were highly heterogeneous not only when dealing with their time frame, but also from a geographic point of view. As previously stressed, the epidemiology of RSV, and particularly its seasonal pattern, have been highly affected and even disrupted by the SARS-CoV-2 pandemic [135,136,137,138,139,140], with a substantial rebound in the following seasons [147,250,251,252]. Even though some studies tentatively countered the mutated epidemiology of RSV through an advanced statistical approach [210], the proper calculation of vaccine efficacy eventually depends on the underlying activity of the condition countered by the assessed immunization. Due to the uneven occurrence of RSV infections in the general population between 2020 and 2023, all estimates on vaccine efficacy from RCTs performed during and immediately after the SARS-CoV-2 pandemic should be accurately and cautiously appraised.

**Figure 3:**
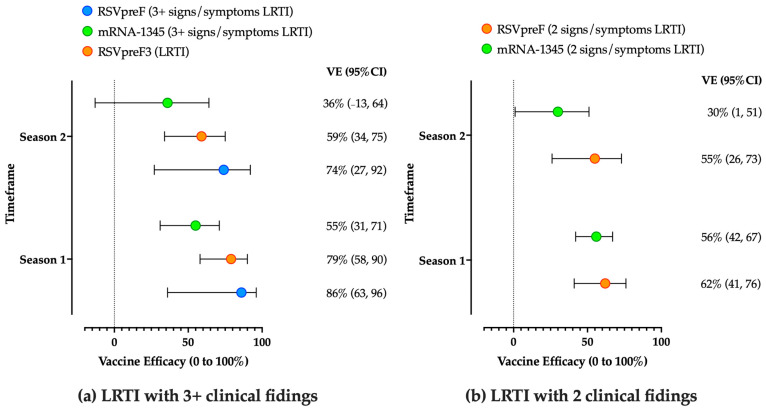
Summary of Vaccine Efficacy with corresponding 95% Confidence Intervals (95%CI) in the prevention of lower respiratory tract illnesses (LRTI) with three or more findings (**a**) and with two clinical findings (**b**) during the first and second respiratory syncytial virus (RSV) season in adults ≥ 60 years [28,87,210,213,221,241,242,243].

Considering all of the aforementioned shortcomings, corresponding estimates for vaccine efficacy calculated from available RCTs were summarized in Figure 3 and Table 2.

Taking into account the aforementioned shortcomings, estimates summarized in Figure 3 appear as extensively overlapped, and no substantial differences could be noticed at the end of the first season after the delivery of the RSV vaccine [244,253,254].

A further shortcoming shared by all available RCTs and stressed by Melgar et al. [232,237] was the lack of sufficient statistical power to ascertain the efficacy of the included vaccines in averting RSV-related hospitalizations and deaths, particularly in older adults (i.e., aged 80 years or older), impairing the extensive generalizability of collected results. This specific knowledge gap was recently filled by preliminary data on hospitalizations in US adults vaccinated against RSV during the RSV season 2023–2024 [255]. Notably, the authors did not report distinctive estimates for both available vaccines (i.e., RSVpreF3 and RSVpreF). Nonetheless, a pooled vaccine efficacy against RSV-associated hospitalization of 75% (95%CI 50 to 87) was calculated for the whole population of adults aged 60 years and more, similar for adults aged 60 to 74 years (75%, 95%CI 31 to 91) and 75 years and older (76%, 95%CI 40 to 91) [255].

**Figure 4:**
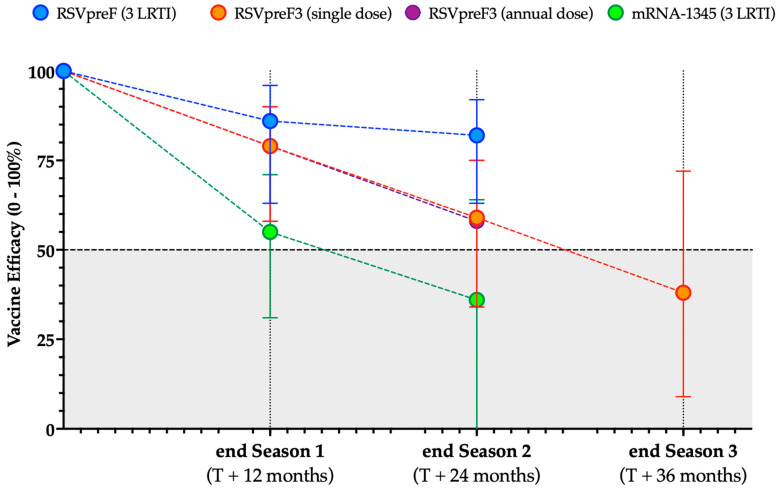
Summary of the decline of Vaccine Efficacy (reported with their corresponding 95% Confidence Intervals [95%CI]) in the prevention of lower respiratory tract illnesses (LRTI) three or more findings during the first and second respiratory syncytial virus (RSV) season [28,87,210,213,221,241,242,243,257]. Preliminary data on season 3 of RSVpreF3 was retrieved from the ACIP Meeting of 24 October 2024 [258].

Even though both ACIP and STIKO have to date recommended a single lifetime dose of the RSV vaccines, a main issue emerging from available RCTs is the duration of the vaccine effectiveness after the primary shot [28,256]. According to the original vaccination schedules assessed during the RCTs, RSVpreF, RSVpreF3, and mRNA-1345 should be delived as a single dose in a lifetime, not requiring annual shots. However, as shown in Figure 4, all vaccines show a certain reduction in the estimated efficacy against LRTI.

An alternative schedule was assessed for RSVpreF3 [210], with a total of 4991 subjects receiving a second dose at the end of the first RSV season. While the vaccine efficacy across season 1 and 2 was estimated to be 78% (95%CI 68 to 85), estimates for season 2 alone were 59% (95%CI 34 to 75) for the conventional schedule and 58% (95%CI 34 to 75) for the two-doses schedule. As the levels of NAs elicited by the second dose were also lower than those reported after the first shot, a second dose remains to date not recommended [105,244,253,254]. Preliminary data on season 3 reported during the ACIP meeting held during the month of October 2024 identified a vaccine efficacy of 48.0% (95%CI 8.7 to 72.0) for Season 3, with a cumulative efficacy (season 1 to 3) equal to 67.4% (95%CI 42.4 to 82.7, including the season covariate) [258].

Regarding RSVpreF, vaccine efficacy was estimated to be 79% (95%CI 26 to 94) for mid-season 2, with a provisionary estimate for the end of season 2 equal to 74% (95%CI 27 to 92), as reported by the ACIP [259,260]. In a subsequent report, the eventual vaccine efficacy across both seasons was then estimated as 81.5% (95%CI 63.3 to 91.6) for LRTI with three or more symptoms and 58.8% (95%CI 43.0 to 70.6) for LRTI with two symptoms [257].

More limited evidence is to date available for mRNA-1345. Eventually, data on mRNA-1345 rather hinted at the total efficacy from the mid of RSV season 1 to the mid of RSV season 2, with an estimated efficacy of 63% (95%CI 37 to 78) [243], while using all available follow-up time (median 18.8 months per participant; range 0.5 to 24) estimated efficacy was 47% (95%CI 35 to 57) against RSV-LRTD with two or more symptoms, and 48% (95%CI 28 to 63%) against RSV-LRTD with three or more symptoms [254]. In a more recent appraisal of available data, ACIP provided an estimated vaccine efficacy of 30% (95%CI 1 to 51) for LRTI with two signs/symptoms, compared to 36% (95%CI -13 to 64) for LRTI with three or more signs/symptoms.

All vaccines appear well-tolerated, with an acceptable safety profile, and can be co-administered with other respiratory virus vaccines (i.e., influenza and COVID-19). Recently, ACIP reviewed available data, including preliminary estimates [261], on co-administration of RSV vaccines with standard seasonal influenza vaccines (SIV), adjuvated SIV, high-dose SIV, and with mRNA COVID-19 vaccine [210,224,262,263]. Even though humoral response against influenza A/Darwin H3N2 was slightly reduced in coadministration of RSVpreF3 with adjuvated influenza vaccines (Geometric mean titer 1.23, 95%CI 1.06 to 1.42), and similarly, GMT for mRNA-1345 were somewhat reduced when co-administered with high-dose SIV, the committee still advocates the co-administration as acceptable [264].

Regarding adverse effects, a small number of inflammatory neurologic events, including Guillain–Barré Syndrome (GBS), were observed after RSV vaccination in clinical trials with subunit vaccines and were also reported across the Vaccine Adverse Event Reporting System (VAERS) database (4.4 and 1.8 reports per million administered doses of RSVpreF and RSVpreF3 vaccines, respectively) [264,265]. A subsequent self-controlled case series design study, whose early results were shown during the ACIP meeting of October 2024, suggested a statistically significant elevation in GBS syndrome risk following RSV vaccination with RSVpreF3 + AS01 (incident rate ratio [IRR] 2.46, 95%CI 1.19 to 5.08), and a statistically non-significant elevation in GBS risk following the delivery of RSVpreF vaccine (IRR 2.02, 95%CI 0.93 to 4.40) [266]. However, the number of incident cases of GBS remains small (i.e., less than 10 cases per 1 million vaccinations), with an attributable number of excess cases equal to 7 cases per million doses (95%CI 2 to 11) for RSVpreF3, and 9 cases per million doses (95%CI 0 to 18) for RSVpreF, comparable to other vaccinations (e.g., SIV, and recombinant zoster vaccine) [265,266,267,268,269,270].

#### 3.2.2. Maternal Vaccination

To date, neither RSVpreF3 nor RSVpreF can be used for immunizing infants and/or children [232,237,271]. However, both vaccines were originally designed to be delivered in pregnant women.

Maternal immunization is an established option to protect young infants from severe infection. The transplacental transfer of natural RSV NA [272,273], and the vaccine-elicited Nas, is both robust and efficient even in low-birth-weight neonates, promising to be quite effective during the early months of life [274]. Still, maternal vaccination against RSV was extensively debated. On the one hand, it is important to stress that only RSVpreF was effectively approved by regulators (e.g., FDA and EMA) [275] for the use as a maternal vaccine. On the contrary, safety issues were raised on RSVpreF3, due to an increased risk of preterm birth observed in the intervention arm compared with the control arm in a phase 3 clinical trial (relative risk 1.37, 95%CI 1.08 to 1.74, *p* = 0.001), urging GSK to suspend the recruitment of new cases during the phase 3 study (RCT NCT04605159) [276,277]. Even though a large RCT on maternal vaccination with RSVpreF neither showed any increase in the rate of preterm birth among vaccinated pregnant women nor in the rate of other adverse neonatal outcomes [278], and despite their different design, due to a small but not-significant increase of preterm birth (around 1%), the FDA and the ACIP recommended the administration of RSVpreF vaccine between 32–36 weeks for a seasonal vaccination against RSV (i.e., September to January) [253,277,279]. Similar recommendations were included in the Canadian Immunization Guide [280]. This approach was not shared by the EMA, that still recommends the original 24th to 36th week, and by the Health ministry of Argentina, as the use of RSVpreF was authorized between weeks 32 and 36 [281,282]. Interestingly, both the UK JCVI and the Belgium Superior Health Council (CSS) suggest the RSVpreF should be delivered after the 28th week of gestation, due to the limited reliability of data for cases with vaccine shots performed before the 28th week [283,284].

When dealing with maternal vaccination and the definition of the optimal timing for vaccination, it is important to stress how it could affect the real-world effectiveness of maternal vaccination. In their recent preprint study, Jasset et al. [285] documented significantly lower cord:maternal transfer ratios in mothers vaccinated 2 to 3 weeks and 3 to 4 weeks before delivery than among mother-child couples vaccinated > 6 weeks prior to delivery (0.76 vs. 1.43, *p* = 0.008 and 0.92 vs. 1.43, *p* = 0.03, respectively). Even though antibody levels against RSV were not statistically different between the groups stratified by timing between vaccination and delivery, maternal vaccination performed earlier than in the approved 32nd to 36th week window (i.e., at least 5 weeks prior to delivery), may therefore result in significantly more efficient antibody transfer to the fetus.

**Figure A1:**
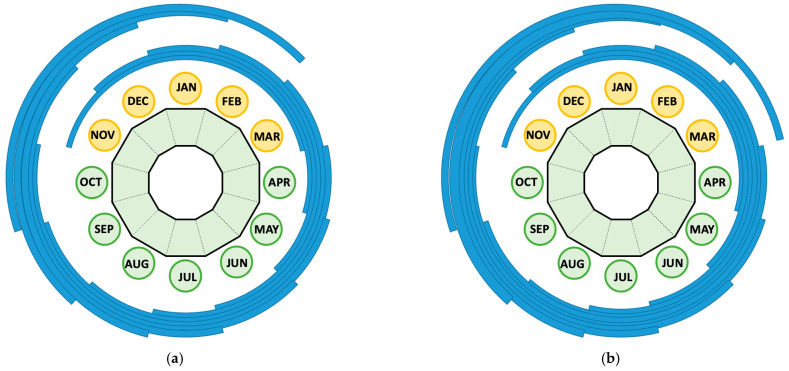
Schematic comparison of the seasonal immunization granted by nirsevimab (5 months after the delivery, subfigure (**a**)) and by the maternal vaccination strategy (6 months after the birth, subfigure (**b**)).

A potentially significant feature of maternal vaccination strategy is that maternal antibodies could guarantee some degree of protection during the first 6 months of life (Appendix A Figure A1). In this regard, an early RCT suggested that RSVpreF may be 57.1% (95%CI 14.7 to 79.8) effective against RSV-associated LRTI 90 days after birth, 56.8% (95%CI 31.2 to 73.4) at 120 days, 52.5% (95%CI 28.7 to 68.9) at 150 days, and 51.3% (95%CI 29.4 to 66.8) at 180 days, while the corresponding vaccine efficacy against medically attended severe LRTI ranges between 81.8% (95%CI 40.6 to 69.3) at 90 days after birth, and 69.4% (95%CI 44.3 to 84.1) at 180 days [286]. Moreover, focusing on RSV-associated hospitalizations, corresponding efficacy would range between 67.7% (95%CI 15 to 89) at 90 days and 56.8% (95%CI 10 to 81) at 180 days. Initial data from Kampmann et al. [286] confirmed by a subsequent subset analysis from Japan hinted at an efficacy against medically attended RSV-LTRI of 100% (95%CI 30.9 to 100) during the first 90 days, and of 87.6% (95%CI 7.2 to 99.7) during the first 180 days [279]. Unfortunately, the sample size (230 vaccinated women and 232 placebo) was too small to provide any reliable estimate on severe medically attended RSV-LTRI. Even though the uptake of the maternal RSV vaccine during the 2023–2024 season was low (according to US estimates, only 18% of the pregnant persons received the vaccine) [277], which somewhat limited early post-licensure studies of efficacy and safety, available data did indicate a rate of pre-term birth at 4.1% that is clearly within the usual background rate, seemingly removing residual concerns for the maternal RSV vaccine. In fact, a recent meta-analysis on the efficacy and real-world effectiveness of RSVpreF as a maternal vaccine confirmed the potential efficacy in reducing hospitalizations of the offsprings of this immunization strategy (RR 0.50, 95%CI 0.31 to 0.82) [287].

### 3.3. Immunization Strategies

Currently, no internationally shared guidelines on RSV immunization strategies were made available. In the following subsections, a detailed analysis of available recommendations for older adults and infant immunization (either by means of mAb or maternal vaccination) will be provided.

#### 3.3.1. Vaccines for Adults

Although all vaccines were proven effective in all adults aged 60 years or older, and nearly all regulatory authorities consider available licensed vaccines for older adults as substantially equivalent in terms of efficacy, effectiveness, and safety profile, leading to a shared blueprint for issued recommendations [237,244,253,254], governing authorities have issued more limited and focused recommendations for heterogenous reasons—most notably, on their documented cost-effectiveness. In fact, a series of recently published studies [288,289,290] are quite consistent in considering RSV vaccination as not cost-effective when administered to the general population aged 60 years or older, with no substantial differences across available formulates. In the subgroup 60 to 64 years, the costs for gained QALY would range between $218,350 for RSVpreF and $372,656 for RSVpreF3, compared to $94,676 for RSVpreF and $167,301 for RSVpreF3 in the age group 65 and more.

Taking into account the available data, including recent modifications in authorization for available vaccines, on 21 June 2023 the United States ACIP recommended that all adults aged ≥ 60 years might receive a single dose of any RSV vaccine (at that time, RSVpreF and RSVpreF3) by means of a shared clinical decision [232,237]. Due to the inappropriate vaccine coverage rates obtained during the first RSV season in individuals characterized by main risk factors for complications of RSV-LRTI, since 26 June 2024 the ACIP has issued a double strategy, recommending a single dose of any available FDA-approved RSV vaccine in all adults aged ≥ 75 years, and in adults aged 60 to 74 years only if reporting underlying chronic medical conditions increasing the risk for RSV-related complications, affected by moderate or severe immunosuppression, or living in nursing homes [254]. More recently, both the ACID and the FDA reviewed the results of a randomized, double-blind, placebo-controlled MONeT trial on the delivery of RSVpreF on 681 adults aged 18 to 59 years affected by chronic conditions potentially leading to more severe clinical outcomes in cases of RSV infection (i.e., chronic pulmonary, cardiovascular, renal, hepatic, neurologic, hematologic, or metabolic disorders; ClinicalTrials.gov Identifier: NCT05842967). As study participants exhibited similar RSV-neutralizing antibody titers, compared with immunocompetent adults aged ≥ 60 years from Pfizer’s main phase 3 trial, the FDA approved the use of RSVpreF even in adults aged 18 to 59 years who are at increased risk for RSV-associated LRTI [240]. Also, RSVpreF3 has recently benefited from an extension of its official indication, being authorized for adults aged 50 to 59 years affected by chronic medical conditions increasing their risk for developing medical complications due to LRTI [238].

Notably, UK JCVI only recommended RSV vaccination for adults aged 75 years or more, not considering cost-effective vaccination of the age group 60 to 74 years, even in cases affected by comorbidities [283]. On the other hand, on 8 August 2024 German STIKO also recommended RSV vaccination in all adults aged 75 years or older, rather suggesting the delivery of either RSVpreF or RSVpreF3 as equivalent options to all adults aged 60 to 74 years in whom RSV infection could modify the course of the underlying disorders, including those living in long-term care centers [244]. Notably, mRNA-1345 was not included in the health technology assessment from STIKO, being not approved by the EMA at the time of the analyses (January 2024). As summarized in Table 3, the approach from ACIP and STIKO was shared by several governing authorities, including those in Australia, Canada, France, Belgium, and Sweden [105,291,292]. In the aforementioned countries, RSV vaccination is therefore recommended in all adults aged 75 years or older, and in the age group 60 to 74 years only in subjects affected by underlying medical conditions that could be associated with high rates of RSV-related complications, hospitalization, and/or death. On the contrary, governing authorities from Austria, Ireland, and Norway provided a quite different strategy. In Austria, RSV vaccination is currently recommended in all adults aged 60 years or more, irrespective of their baseline health status [293], and in adults aged 18 years or more with underlying medical conditions. In Ireland, the delivery of either RSVpreF or RSVpreF3 is recommended in all adults older than 65 years, with and without underlying medical conditions, while Norwegian guidelines only recommended RSV vaccines for subjects older than 60 years in whom RSV infection would increase the risk for severe disease and/or complications of an underlying medical condition [294,295].

Interestingly, even those that are considered relevant medical conditions are affected by significant heterogeneity across the available guidelines. For example, USA ACIP, Canadian National Advisory Committee on Immunizations, STIKO, and Austrian guidelines include a detailed list of relevant medical conditions [253,254,293,296], while Norway preferred a more general approach, addressing the medical conditions or risk factors that a health care provider determines would increase the risk for severe disease due to viral respiratory infection [295]. Moreover, even where a detailed list was provided, individual risk factors were characterized by an inconsistent definition. For instance, ACIP in the USA and NACI in Canada recommend RSV vaccine only for adults aged 60 to 74 years-old with a BMI ≥ 30 kg/m^2^; Australian recommendations include only severe obesity (i.e., BMI > 40 kg/m^2^). Similarly, Canadian and USA recommendations include chronic liver disease, which is otherwise reported only by Swedish guidelines, and while Belgium, Sweden, and Australia include diabetes mellitus as a risk factor irrespective of its functional status, USA and Germany only limit their recommendations to complicated diabetes mellitus.

#### 3.3.2. Immunization Strategies for Infants and Small Children

Compared to RSV vaccination in adults, choosing the most appropriate immunization strategies for infants and small children appears as way more tantalizing, for several reasons.

First and foremost, it is important to stress that while a growing amount of evidence was published on the effectiveness of nirsevimab in averting RSV-associated LRTI, results of real-world studies on maternal vaccination strategies may be available in the near future. Second, according to available data, strategies are equally cost-effective. As stressed by Guinazú et al. [13], implementing either RSV-prevention strategy could prevent a substantial number of RSV cases, medical consultations, hospital admissions, and even RSV-related deaths, with no substantial differences and comparable healthcare costs savings (around 54 million US dollars for mAb, 47.4 million dollars for maternal RSV vaccine). According to national estimates from Argentina, and from a 10-years’ perspective, it means around 500,000 RSV cases (−620,601 vs. −431,589 by means of mAb and maternal vaccination, respectively), around 400,000 clinic visits (-461,609 vs. −324,429), 60,000 RSV hospital admissions (66,921 vs. 61,633), and over 1000 RSV-related deaths (1451 vs. 1313).

Even though these data are in line with preliminary estimates provided before the licensing of both nirsevimab and maternal vaccines [299,300,301], it must be noted that both options have substantial pros and cons (Table 4). Their cost-effectiveness is heavily dependent on two distinctive factors: the costs associated with the preventative intervention (i.e., vaccines and/or the mAb) and the costs of medical care associated with RSV cases.

Unsurprisingly, the current international indications are highly heterogenous. For example, according to the Canadian Immunization Guide [280], the Austrian Guidelines [293], and the Health Council of the Netherlands, nirsevimab should be prioritized over RSVpreF when possible [302], but in all of the aforementioned countries RSVpreF is still considered a potential, individual option when the expected date of delivery occurs shortly before or during the next RSV season. On the contrary, ACIP recommendations [254], as well as the independent statement from JCVI [283], and the recommendations from Belgium CSS [284], report no preferences between mAb and the maternal vaccine over the choice of preventative intervention during the first year of life.

Interestingly, maternal vaccination strategy and mAbs are increasingly emerging as a potentially integrated preventative approach. For example, the American Academy of Pediatrics recommends that nirsevimab be delivered to infants born from vaccinated women when there is the reasonable doubt about the mounting of adequate immune response to vaccination (i.e., underlying immunocompromising conditions) or any underlying condition that may have reduced the transplacental antibody transfer (e.g., people with HIV infections; infants who have undergone cardiopulmonary bypass or extracorporeal membrane oxygenation), or are at substantially increased risk for severe RSV disease (i.e., those with a previous indication for being treated with palivizumab) [303]. Moreover, nirsevimab should be delivered to infants and children 8 through 19 months of age considered at increased risk for RSV disease and entering their second RSV season, including those previously recommended to receive palivizumab regardless of the vaccination status of the pregnant parent.

#### 3.3.3. Timing of Immunization

As for general guidelines, no general guidelines on the timing for the delivery of vaccines and mAb have been provided and shared. In fact, local features of RSV epidemiology urge for accurately tailoring the timing for the delivery of vaccination options, taking into account the different coverage granted by maternal vaccines (6 months after the birth), and mAb (5 months after the delivery for nirsevimab, and 1 month after each dose for palivizumab) (See Appendix A Figure A1).

**Figure 5:**
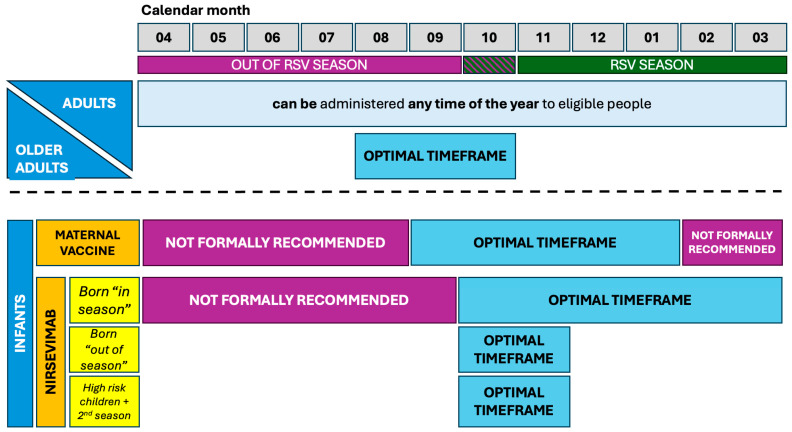
Summary of current recommendations for the delivery of RSV preventive measures in adults and children, as provided by the United States CDC and ACIP [237,304,305,306].

Still, the United States Centers for Disease Control and Prevention and ACIP have provided a series of recommendations that may represent a sort of blueprint for most countries from the Northern Hemisphere, which are summarized in Figure 5.

Briefly, in adults and older adults, RSV vaccines can be administered any time of the year to eligible people, with an optimal time frame ranging from August to October (i.e., immediately before the onset of RSV season) [306].

Protection of infants may be achieved by either maternal vaccine, which should be delivered in women during weeks 32 through 36 of pregnancy, sometime between September and January; delivery is not recommended outside of this window [305]. As most European countries do not share the current recommendations about the time frame of maternal vaccination, with a vaccination window between the 24th and the 36th week, the optimal time frame should be revised to guarantee the maximal protection for children with a presumptive date of birth during the RSV season.

Finally, immunization achieved by means of nirsevimab identifies an optimal time frame between the months of October and March (ideally administered immediately before the hospital discharge) for children born during RSV season, while the optimal time frame for children born out of season and during their second season (i.e., high-risk children) is shortly before the peak of RSV season (i.e., October or November) [304].

## 4. Conclusions

Available data suggest that vaccination of older adults (i.e., ≥75 year or age) with any licensed RSV vaccine may represent a safe and effective intervention, able to reduce the total burden of RSV-related complications. Similarly, vaccination of adults aged 60 to 74 years old could be suggested to all individuals with any underlying condition who may experience severe complications due to RSV infections (i.e., cardiovascular diseases, chronic respiratory diseases). Even adults 18 to 59 may benefit from RSV vaccine if affected by chronic conditions increasing the risk for complicated LRTI. In the next RSV seasons, effectiveness data on RSV vaccines and mAb will be useful for designing and promoting the appropriate immunization strategies, including booster doses and appropriate timing for revaccination according to the waning of vaccine efficacy.

Despite the documented efficacy of both available strategies (i.e., mAb and maternal immunization), more conflicting conclusions could be drawn for immunization of children and infants. As the potential effectiveness of maternal vaccination and the delivery of mAb may be considered as highly comparable, the eventual choice should rely on a public health-oriented approach, finalized to the optimization of available assets. For example, in countries where maternal vaccination has been established as a reliable and effective intervention, maternal RSV vaccination may protect all children born shortly before or during the RSV season. It is important to stress that the most valuable asset associated with RSV maternal vaccination is that the newborn will be protected against RSV since birth and during the first months of life. Unfortunately, when the birth occurs shortly after the end of RSV season, or when the administration of the vaccine is too close to delivery, maternal vaccination would be unable to provide maximum protection. Therefore, in countries where maternal vaccination rates still experience significant constraints, and for individual cases not potentially benefiting from the vaccination, nirsevimab and future mAbs may provide a reliable option for protecting infants and children during RSV season, not only during the first year but also during the second year of life and possibly afterwards. In this regard, it is important to stress that while maternal vaccination can be performed with limited constraints only due to the vaccination window, the delivery of mAb must be performed in a controlled environment, i.e., a vaccination clinic and service, sharing spaces and time slots with other planned vaccinations, contributing to the consumption of available resources. As a consequence, mAb and maternal vaccination should be considered as complementary rather than alternative options.

## Figures and Tables

**Table 1:** The main characteristics of licensed respiratory syncytial virus (RSV) vaccines and their corresponding current approval according to the FDA [238,239,240].

Vaccine	Characteristics	Age Groups	Reference
Arexvy RSVPreF3	Monovalent (RSV A) stabilized prefusion F protein (120 µg) Adjuvant: AS01 Single dose	49–59 years (chronic medical conditions) 60–74 years (chronic medical conditions) ≥75 years (all)	[210,241,242]
Abrysvo RSVpreF	Bivalent (RSV A and B) stabilized prefusion F protein (60 µg + 60 µg); Adjuvant: none Single dose	18–59 years (chronic medical conditions) 60–74 years (chronic medical conditions) ≥75 years (all) Maternal use (all age groups)	[87,221]
mRESVIA mRNA-1345	Monovalent (RSV A) Single mRNA sequence encoding for a stabilized prefusion F protein (50 µg) Adjuvant: none Monovalent	60–74 years (chronic medical conditions) ≥75 years (all)	[213,243]

**Table 2:** Summary of Vaccine Efficacy with corresponding 95% Confidence Intervals (95%CI) in preveting lower respiratory tract illnesses (LRTI) with two and three or more findings during the first and second respiratory syncytial virus (RSV) season [28,87,210,213,221,241,242,243].

Vaccine	Primary Outcome	Efficacy (Point Estimate, 95%CI)	
		First RSV Season (0 to 12 Months)	Second RSV Season (13 to 24 Months)
RSVpreF	LRTI with two signs/symptoms	62% (41, 76)	55% (26, 73)
	LRTI with ≥three signs/symptoms	86% (63, 96)	74% (27, 92)
RSVpreF3, single dose	LRTI ^a^	79% (58, 90)	59% (34, 75)
RSVpreF3, two doses	LRTI ^a^	-	58% (34, 75)
mRNA-1345	LRTI with two signs/symptoms	56% (42, 67)	30% (1, 51) ^b^
	LRTI with ≥three signs/symptoms	55% (31, 71)	36% (−13, 64) ^b^

a = definition of LRTI in randomized controlled trials assessing the Vaccine Efficacy of RSVpreF3 required ≥ two lower respiratory symptoms or signs (including ≥ one sign), or ≥three lower respiratory symptoms. b = mean follow-up per participant, 7 months.

**Table 3:** Summary of the main international recommendations for RSV vaccination in older adults (Note: COND. = conditioned; AUTH. = Authorized).

	USA [253,254]	Canada [296]	UK [297]	Germany [244]	France [105]	Austria [293]	Belgium [291]	Sweden [292]	Ireland [294]	Norway [295]	Australia [298]
**Indication by age**											
18–59 years	AUTH.	NO	NO	NO	NO	COND.	NO	NO	NO	NO	NO
60–64 years	COND.	COND.	NO	COND.	COND.	ALL	COND.	COND.	NO	COND.	COND.
65–74 years	COND.	COND.	NO	COND.	COND.	ALL	COND.	COND.	ALL	COND.	COND.
≥75 years	ALL	ALL	ALL	ALL	ALL	ALL	ALL	ALL	ALL	COND.	ALL
**High-risk groups comorbidities**											
Chronic cardiovascular disease	X	X		X	X	X	X	X			X
Chronic lung or respiratory disease	X	X		X	X	X	X	X			X
Chronic renal disease		X				X	X	X			X
End-stage renal disease	X	X		X		X	X				
Uncomplicated diabetes mellitus		X					X	X			X
Complicated diabetes mellitus	X	X		X			X	X			X
Any neurologic or neuromuscular conditions											X
Neurologic or neuromuscular conditions causing impaired airway clearance or respiratory muscle weakness	X	X		X		X					
Chronic liver disease	X	X						X			
Neoplasia						X					
Chronic hematologic conditions	X			X		X	X				
Obesity (BMI ≥ 30 kg/m^2^)											X
Severe obesity (BMI ≥ 40 kg/m^2^)	X	X				X	X				
Any immune compromise	X	X				X	X	X			X
Severe immune compromise	X	X		X		X	X	X			
Residence in a nursing home	X	X		X		X	X	X			
Chronic medical conditions or risk factors that a health care provider determines would increase the risk for severe disease due to viral respiratory infection	X									X	

**Table 4:** Comparison between immunization strategies based on maternal vaccination and monoclonal antibodies.

	Maternal Vaccination	Monoclonal Antibodies
**Advantages**	Not requiring shots to the newborns.	Can be delivered any time up to 2 years of age.
	Effective protection guaranteed from birth up to 180 days.	Effective protection expected up to 150 days after the delivery.
	Potential resistance to preF mutations solicited by nirsevimab.	Likely well-accepted by parents from countries with high pediatric vaccination rates.
**Disadvantages**	Transplacental transfer of maternal antibodies depends on the production of IgG-class neutralizing antibodies: it could be insufficient due to maternal conditions (e.g., any immunodeficiency status), or premature delivery.	Requiring shots to the newborns.
	Doubts on its efficacy in pre-term infants (even if delivered in optimal maternal:foetus transfer windows).	High cost of conventional mAb (serial shots during the winter season)
	Possibly affected by general vaccine hesitancy for maternal vaccination (likely heterogeneous acceptance in various countries).	Potential emergence of mutated strains due to selective pressure of the monoclonal antibody.

## Data Availability

Not applicable.
