# Correction: Riccò et al. Respiratory Syncytial Virus: A WAidid Consensus Document on New Preventive Options. *Vaccines* 2024, *12*, 1317

**DOI:** 10.3390/vaccines13080878

**Published:** 2025-08-20

**Authors:** Matteo Riccò, Bahaa Abu-Raya, Giancarlo Icardi, Vana Spoulou, David Greenberg, Oana Falup Pecurariu, Ivan Fan-Ngai Hung, Albert Osterhaus, Vittorio Sambri, Susanna Esposito

**Affiliations:** 1 Servizio di Prevenzione e Sicurezza Negli Ambienti di Lavoro (SPSAL), AUSL-IRCCS di Reggio Emilia, Via Amendola 2, 42122 Reggio Emilia, Italy; matteo.ricco@ausl.re.it; 2 Canadian Center for Vaccinology, Dalhousie University, IWK Health Centre and the Nova Scotia Health Authority, Halifax, NS B3K 6R8, Canada; bh723616@dal.ca; 3 Departments of Pediatrics, Dalhousie University, Halifax, NS B3K 6R8, Canada; 4 Departments of Microbiology and Immunology, Dalhousie University, Halifax, NS B3H 4R2, Canada; 5 Department of Health Sciences (DISSAL), University of Genoa, 16132 Genoa, Italy; icardi@unige.it; 6 IRCCS Ospedale Policlinico San Martino, 16132 Genoa, Italy; 7 Immunobiology and Vaccinology Research Laboratory and Infectious Diseases Department “MAKKA”, First Department of Paediatrics, “Aghia Sophia” Children’s Hospital, Athens Medical School, 11527 Athens, Greece; vspoulou@med.uoa.gr; 8 Pediatric Infectious Diseases Unit, Soroka University Medical Center, Faculty of Health Sciences, Ben Gurion University, Beer Sheva 8410501, Israel; dudi@bgu.ac.il; 9 Children’s Clinical Hospital Brasov, 500063 Brasov, Romania; oanafp@yahoo.co.uk; 10 Faculty of Medicine Brasov, Transilvania University, 500019 Brasov, Romania; 11 Division of Infectious Diseases, Department of Medicine, Queen Mary Hospital, The University of Hong Kong, Hong Kong SAR 999077, China; ivanhung@hku.hk; 12 Research Center for Emerging Infections and Zoonoses, University of Veterinary Medicine Hannover, 30559 Hannover, Germany; albert.osterhaus@tiho-hannover.de; 13 Unit of Microbiology, The Greater Romagna Area Hub Laboratory, 47522 Cesena, Italy; vittorio.sambri@unibo.it; 14 Department Medical and Surgical Sciences (DIMEC), Alma Mater Studiorum University of Bologna, 40126 Bologna, Italy; 15 Pediatric Clinic, Department of Medicine and Surgery, University of Parma, 43126 Parma, Italy

The authors would like to make the following corrections to this published paper [1]. In the original publication, there was a mistake in Figure 4 as published. Factual inaccuracies in Figure 4 were corrected. In fact, actual data do not support difference in efficacy between the respective vaccines as the primary endpoints from the clinical trials have been defined differently, and therefore no comparison between vaccines is appropriate, also taking into account the heterogeneous follow up during RSV seasons. The corrected Figure 4 appears below. The authors state that the scientific conclusions are unaffected. This correction was approved by the Academic Editor. The original publication has also been updated.

## Figures and Tables

**Figure 4:**
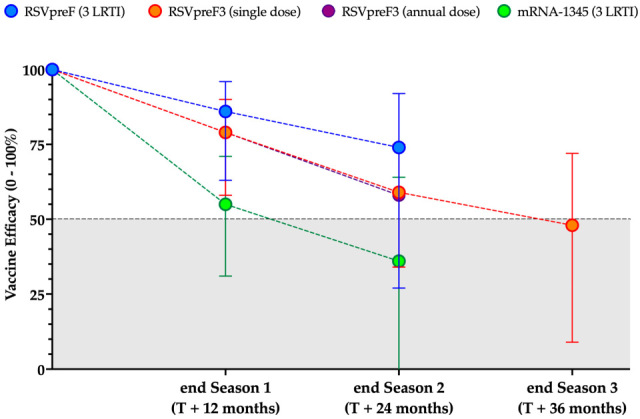
Summary of the decline in vaccine efficacy (reported with their corresponding 95% Confidence Intervals [95%CI]) in the prevention of lower respiratory tract illnesses (LRTI) with 3 or more findings during first and second respiratory syncytial virus (RSV) season [28,87,210,213,221,241,243,258]. Preliminary data on Season 3 of RSVpreF3 have been retrieved from ACIP Meeting of 24 October 2024 [259]. The figure is not intended for vaccine comparison and in particular, the x axis does not represent a defined timeline, but seasonal efficacy only.
